# Diverse cloud radiative effects and global surface temperature simulations induced by different ice cloud optical property parameterizations

**DOI:** 10.1038/s41598-022-14608-w

**Published:** 2022-06-22

**Authors:** Bingqi Yi

**Affiliations:** 1 School of Atmospheric Sciences and Guangdong Province Key Laboratory for Climate Change and Natural Disaster Studies, Sun Yat-sen University, Guangzhou, 510275 China; 2 Southern Marine Science and Engineering Guangdong Laboratory (Zhuhai), Zhuhai, 519082 China

**Keywords:** Climate sciences, Environmental sciences

## Abstract

The representation of ice cloud optical properties in climate models has long been a difficult problem. Very different ice cloud optical property parameterization schemes developed based on very different assumptions of ice particle shape habits, particle size distributions, and surface roughness conditions, are used in various models. It is not clear as to how simulated climate variables are affected by the ice cloud optical property parameterizations. A total of five ice cloud optical property parameterization schemes, including three based on the ice habit mixtures suitable for general ice clouds, mid-latitude synoptic ice clouds, and tropical deep convective ice clouds, and the other two based on single ice habits (smooth hexagonal column and severely roughened column aggregate), are developed under a same framework and are implemented in the National Center for Atmospheric Research Community Atmospheric Model version 5. A series of atmosphere-only climate simulations are carried out for each of the five cases with different ice parameterizations. The differences in the simulated top of the atmosphere shortwave and longwave cloud radiative effects (CREs) are evaluated, and the global averaged net CRE differences among different cases range from − 1.93 to 1.03 Wm^−2^. The corresponding changes in simulated surface temperature are found to be most prominent on continental regions which amount to several degrees in Kelvin. Our results indicate the importance of choosing a reasonable ice cloud optical property parameterization in climate simulations.

## Introduction

Ice clouds distribute globally and exert remarkable impacts on climate^1–5^. Because of the intricate complexities in ice cloud, it remains a big challenge to accurately calculate the optical properties of ice clouds and subsequently well simulate the ice cloud radiative impacts^6,7^. The complexity of ice cloud problem lies in its versatile of ice habits, wide range of ice particle sizes, and various degrees of ice surface roughness as well as internal inhomogeneity^8–11^.

As a result, selecting a valid ice cloud particle shape model for remote sensing and climate modeling studies became one of the most important but difficult questions. Many studies endeavored to seek for an appropriate solution, for example, spherical ice particle approximation and various non-spherical ice particle habits were used in previous ice cloud property parameterizations^12–23^. A frequently used ice cloud particle model is the single hexagonal column with smooth surface^24^. Ice habit mixtures are also proposed and used in a couple of studies. For example, the ice cloud model for MODIS (Moderate Resolution Imaging Spectroradiometer) collection 5 cloud property retrieval^25^, the general ice habit mixture model^26^, the two-habit model^27^, and various other^28–35^. Advances in the calculations of the optical properties of ice particles were summarized in more details in Baran^36,37^ and Yang et al.^9^. The MODIS science team however adopted a single-habit ice cloud particle model for the MODIS Collection 6 ice cloud retrieval algorithm^2^ (hereafter, C6 ice cloud model). The C6 ice cloud model is a severely roughened hexagonal column aggregate that encompasses the non-spherical characteristics and surface roughness features of real ice cloud particles, as compared to the ice cloud particle model of MODIS Collection 5. It is not only implemented in the latest MODIS cloud product retrievals but also incorporated into fast radiative transfer models and significantly improves the ice cloud modeling capabilities for various satellite sensors^38,39^. Yi et al.^40,41^ also applied the C6 ice and liquid water cloud models in the broadband radiative transfer models, and achieved better simulation performances in deriving the top of the atmosphere (TOA) cloud radiative effect (CRE) in comparison with Clouds and the Earth’s Radiant Energy System (CERES) observations. Although some studies^42,43^ found that the C6 ice cloud model exhibited inconsistent performances in the mid and far infrared regions, it generally provided reasonable results for broadband simulations. Apparently, the present status of implementing various kinds of ice cloud optical property parameterizations in remote sensing and modeling studies could induce large uncertainties in the applications^32^.

The model simulated climate states and variables are closely related to how the ice cloud optical properties are parameterized and how large the CREs exert on the climate system. The cloud physics and radiation process modeling in general circulation models still contained a lot of uncertainties in comparison with observations^44–46^. Yi et al.^47^ explored the influence of ice particle surface roughening on the global cloud radiative effect. They estimated a large shortwave (SW) globally averaged CRE of 1–2 Wm^−2^ and a small but non-negligible longwave (LW) CRE due to ice crystal surface roughness. Regional CRE differences could be even larger and possibly exerted noticeable impacts on simulated climate. In the comprehensive review by Yang et al.^9^, they compared two climate simulations with assumptions of spherical and non-spherical ice cloud optical parameterizations. They derived a generally warmer climate especially over land with spherical ice cloud assumption. Baran et al.^32,48^ found the impacts of coupled cloud physics-radiation parameterization on general circulation models could be quite significant. Zhao et al.^49^ reported large differences in the simulated CREs, vertical heating rate, precipitation, and atmospheric circulation among various model runs with the Community Earth System Model (CESM) default^29^, Fu^14^, and Baum-Yang^26^ (the GHM case in this study) ice optical properties. Järvinen et al.^50^ replaced the ice optical properties used in the ECHAM–Hamburg Aerosol Module (ECHAM-HAM) general circulation model with those of a new parameterization and found that the complex ice crystal case exerted an additional cooling effect of 1.12 Wm^−2^ on the global radiation budget. However, such comparison studies are often based on very different ice parameterizations. Not only the ice habits and particle size distribution are different, but also many other factors including the ice refractive indices, the treatment of ice surface roughness conditions, and the definitions of ice cloud effective diameter (radius), are interfering the overall results of the simulated CREs and the climate states. In other words, the induced changes in simulated climate states and variables could be biased in previous studies. Note Baran et al.^32^ made significant efforts to explore the inconsistencies of assumptions involved in the cloud physics and radiation without using the concept of effective diameter, which shed some light on solving this problem from another angle. This study aims at evaluating and quantifying the impacts of various ice cloud particle models developed under a same framework with considerations of different degrees of complexities to constrain the uncertainties associated with ice cloud habit models.

## Results

### Ice cloud scattering property parameterizations

**Figure 1:**
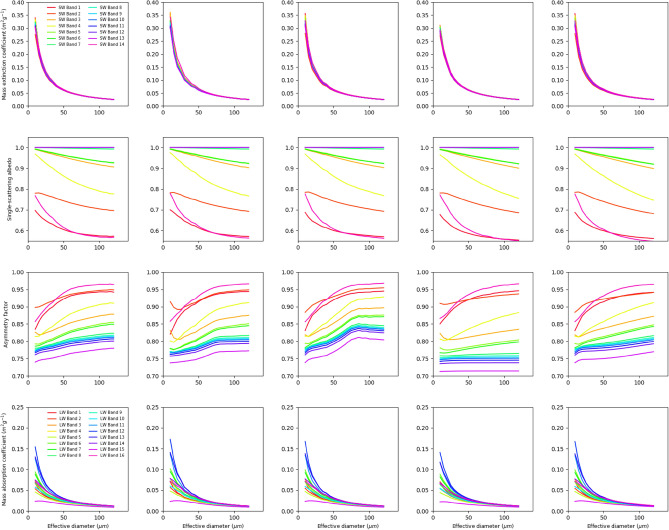
Ice cloud optical properties parameterized as functions of the ice particle effective diameter for various SW and LW bands. Rows from top to bottom are the mass extinction coefficient, single-scattering albedo, asymmetry factor, and mass absorption coefficient. Columns from left to right are the GHM, MLC, TDC, ASC, and SCN ice habit cases. The spectral ranges of SW and LW bands are listed in Table 2.

Figure 1 shows the five sets of ice cloud optical property parameterization schemes developed based on the mixed-habit ice particle models of General Habit Mixture (GHM), Midlatitude habit mixture (MLC), and Tropical Deep Convection mixture (TDC), as well as two single-habit ice cloud particle models, namely, Aggregate of Severely roughened column (ASC) and Single Column (SCN) (see also Table 1 for the abbreviations of the five cases and Supplementary Fig. 1 for the differences of various ice scattering properties between the GHM and the other 4 schemes). The variables shown in Fig. 1, namely mass extinction coefficient, single-scattering albedo, and asymmetry factor for the shortwave and mass absorption coefficient for the longwave, together with the prognostic/diagnostic cloud microphysical properties (i.e., cloud optical depth and cloud effective particle size), are the properties needed for the models to calculate cloud radiative effects. The wavelength ranges of radiation bands are listed in Table 2.

**Table 1:** Explanations of abbreviations for the five ice cloud models.

	Explanations of ice cloud models
GHM	General habit mixture model: developed based on all ice cloud observations in field campaigns globally; suitable for global ice cloud particle representation
MLC	Midlatitude habit mixture model: developed based on ice cloud observations in field campaigns at midlatitude regions; suitable for midlatitude ice cloud particle representation
TDC	Tropical deep convection habit mixture model: developed based on ice cloud observations in field campaigns at the tropics; suitable for tropical ice cloud particle representation
ASC	Aggregate of severely roughened hexagonal column model: adopted by MODIS collection 6 cloud retrieval algorithm^2^ to retrieve ice clouds globally
SCN	Single hexagonal column model: adopted by various radiation modules for ice cloud radiative transfer modeling (note detail aspect ratios of hexagonal column may vary in different applications, i.e., Ebert and Curry^12^; Fu et al.^13,14^)

**Table 2:** The shortwave and longwave bands of the radiative transfer module of CESM/CAM5 (RRTMG).

Shortwave bands	Wavelength range (μm)	Longwave bands	Wavelength range (μm)
1	0.2–0.26	1	3.077–3.846
2	0.26–0.34	2	3.846–4.202
3	0.34–0.44	3	4.202–4.444
4	0.44–0.63	4	4.444–4.808
5	0.63–0.78	5	4.808–5.556
6	0.78–1.24	6	5.556–6.757
7	1.24–1.3	7	6.757–7.194
8	1.3–1.63	8	7.194–8.475
9	1.63–1.94	9	8.475–9.259
10	1.94–2.15	10	9.259–10.204
11	2.15–2.5	11	10.204–12.195
12	2.5–3.08	12	12.195–14.2857
13	3.08–3.85	13	14.2857–15.873
14	3.85–12.2	14	15.873–20.00
		15	20.00–28.571
		16	28.571–99.0

It is found that the mass extinction coefficient exponentially decreases with the increase of effective particle diameter at all radiation bands. The most prominent differences between the GHM and the other four cases exist when the effective diameter is under 60 μm, while almost no difference in mass extinction coefficient is found for large particles. Except for the ASC case, the MLC, TDC, and SCN cases exhibit larger value of mass extinction coefficients for the smaller particles with effective diameters lower than 60 μm. These results are similar as those of previous studies^47,51,52^.

The single-scattering albedo (SSA) of the GHM is almost a constant for selected SW bands regardless of the effective sizes of the particle while the other bands show decreased SSA as particle sizes increase. This means ice clouds with larger effective diameters become more absorptive than the smaller ones. All four other cases show some perturbations in single-scattering albedo at various particle sizes and bands, but these perturbations are generally small (< 0.03) and negligible.

The asymmetry factor for the GHM generally increases with the increase of effective particle diameter for all SW bands where the increase rate is higher for some bands than the other. That means the forward scattering of radiation gradually strengthens with the increase of effective particle size. The MLC and SCN have very similar variation pattern as the GHM where the differences in asymmetry factor are lower than 0.02. The TDC however has higher asymmetry factor from the visible to near infrared bands where the largest difference exists around effective particle diameter of 70 μm. This is related to the larger fractions of aggregates of plates when the TDC particle size increases, which strengthen the forward scattering. Conversely, the ASC has remarkably lower asymmetry factor than that of the GHM and possesses the lowest asymmetry factor among all cases, because the severely roughened column ice aggregate is highly complex in shape and in surface condition which bring down the forward scattering. As the effective particle size increases, the difference in the asymmetry factor also increases, due to the lower increase rate of asymmetry factor with effective size in the ASC as compared with that of the GHM.

The mass absorption coefficients at the LW bands follow similar exponentially decreasing variation pattern with increasing effective diameters as the mass extinction coefficients at the SW bands, but different LW bands have different decreasing rate. The ASC also has the most distinct feature in that lower mass absorption coefficients are found as compared with those of the GHM at the smaller effective particle sizes.

Generally, the asymmetry factors and the mass extinction/absorption coefficients play the most prominent roles in determining the cloud optical effects. But it is also important that the climate models accurately prescribe or diagnose the ice cloud effective particle diameters from the model inherent cloud microphysical parameterization. And, it is best that the cloud microphysical and radiative parameterizations follow the same definition of effective particle size^40,41,53^. In this study, we find that the diagnosed ice cloud effective diameters in the five simulation cases are similar and no significant changes exist among them. This is verified by examining the model simulated ice cloud physical properties within the Cloud Feedback Model Intercomparison Project Observation Simulator Package (COSP) results (Supplementary Fig. 3). It is found that all the simulations significantly overestimate the ice effective particle diameter (with global average around 140 μm) (Supplementary Fig. 5) in comparison with the MODIS satellite retrieval (global average around 50 μm in collection 5.1 and 60 μm in collection 6)^40^. This means the cloud radiative impacts are largely determined by the ice cloud particles with large effective sizes in this study. This feature is important to help understand how ice cloud optical properties influence the simulated climate states.

### Shortwave and longwave cloud radiative effects

**Figure 2:**
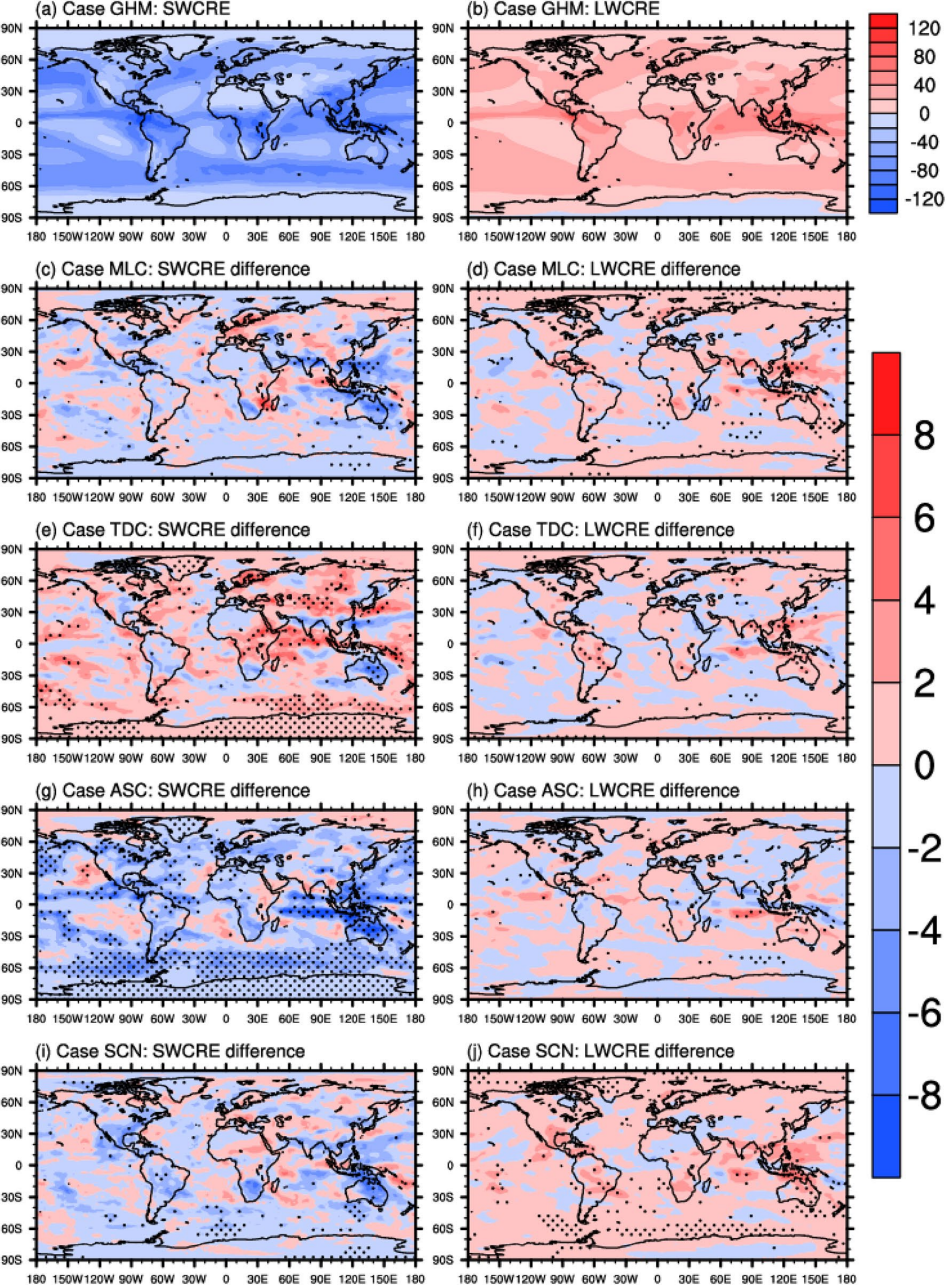
Simulated annual averaged SW and LW cloud radiative effects for the GHM case (**a**, **b**) and the corresponding differences between the MLC, TDC, ASC, SCN and the GHM cases (**c**–**j**). The left column panels are the shortwave CREs and the right column panels are the longwave CREs. Unit: W m^−2^. Stippled areas indicate significant differences at the 95% confidence level.

As mentioned above, the simulated cloud fields in the five sets of simulations are mostly the same (see Supplementary Figs. 3, 4, and 5). Thus, the differences in the TOA CRE among the various cases are largely due to the differences in the ice cloud optical properties depicted in Fig. 1. From Fig. 2 and Table 3, it is apparent that the magnitudes of the SWCRE and LWCRE for the GHM case are close to the previous results using the same model (but with different versions of model)^47^. However, none of the simulations shows similar CREs to CERES Energy Balanced and Filled (Edition 4.1) CREs (Supplementary Fig. 6). The model simulated SWCREs are overestimated in the tropical area and are underestimated in the high latitude region which result in a much stronger globally averaged SWCRE overall. The LWCREs are also overestimated in the tropical oceans while they are underestimated in the mid and high latitudes in the models. The SWCRE and LWCRE of the MLC, TDC, ASC, and SCN cases are compared with the GHM CRE in the following to discern the unique changes in CRE pertaining to each ice parameterization.

**Table 3:** Globally averaged shortwave and longewave TOA cloud radiative effects for the GHM case, as well as the differences between various cases and the GHM. Unit: Wm^−2^.

	GHM	MLC-GHM	TDC-GHM	ASC-GHM	SCN-GHM
SWCRE	− 53.73	− 0.51	0.85	− 2.04	− 0.69
LWCRE	24.21	0.33	0.18	0.11	0.69

The MLC case is the closest resemblance to the GHM case in that the MLC is based on the midlatitude ice cloud habit composition but excludes large aggregates of plates which are frequently found in tropical deep convection. Thus, there is no wonder that the significant differences generally occur over tropical region, for instance, the western north Pacific warm pool region, where larger fractions of ice clouds occur. The globally averaged SWCRE for the MLC case is stronger by about 0.5 Wm^-2^ while the LWCRE is also stronger which results in counteractive effect on the net CRE.

The TDC case, however, shows a very different global map of SWCRE among all the cases in that significantly weaker SWCREs are found not only in the tropics but also in the midlatitude and the polar regions. The weaker SWCRE in general could be largely attributed to the remarkably higher asymmetry factor of the TDC ice particle model. On the contrary, the mass absorption coefficients of the TDC are not very different from those of the GHM, and thus contribute to little differences in the LWCREs.

The strongest global SWCRE is found in the ASC case. The asymmetry factor is significantly reduced when the severely roughened ice hexagonal column aggregate is used as the ice particle model. For several visible bands, the asymmetry factors at the ice effective diameters around 80 μm could be reduced by more than 0.05. Larger perturbations to the SSA are found for the ASC case which indicate some increases in the absorptivity at several ultraviolet and near infrared bands. The mass extinction coefficients also show some reductions at the small effective particle sizes. But the features of the mass extinction coefficient and the SSA of the ASC case are not expected to exert large influence because the ice effective particle sizes simulated in CAM5 are quite large. Thus, the additional shortwave cooling is mainly caused by the reduced asymmetry factor. This is similar as what is found by Yi et al.^47^ and Javanen et al.^50^ Again, minor LWCRE differences are found although apparently lower mass absorption coefficients are found for the ASC case.

The SCN case shows similar SWCRE distribution pattern as that of the MLC while more significant changes are evident over the southern oceans. From Fig. 1, it is also evident that the shortwave bulk scattering properties of MLC and SCN are similar. The most distinct feature of the SCN case is with the LWCRE where positive differences are found over high latitude region. Given the longwave cloud scattering effect is neglected in the RRTMG LW radiation module used in CESM1/CAM5, the LWCRE is solely determined by the mass absorption coefficient and the effective particle size. As a result, the slightly larger mass absorption coefficient at the larger effective particle size could be responsible for the different features of LWCRE distribution.

The differences in the CREs among the five cases can be somehow assessed quantitatively in this study to identify the impacts of factors related to the ice cloud particle models. For example, in comparing among the ice habit mixture particle models like the GHM, MLC, and TDC where various ice habits have different fractions, it is evident that considering certain ice habits (i.e., aggregates of plates) will perturb the SWCRE and LWCRE differently. This means using the ice cloud models suitable for certain regions to represent the global ice clouds could be problematic. According to the previous studies^36,54^, the asymmetry factor for some shortwave bands should be within 0.75 $\pm$ 0.2, which is depicted well by the ASC model. In comparison with the SCN model, the particle shape and surface roughness effects of the ASC contribute to stronger increase in the globally averaged SWCRE on the order of 1 ~ 1.5 Wm^−2^, but not necessarily larger. The large biases between the model CREs and the CERES CREs are mostly attributed to the defects in the cloud microphysical process parameterization in the CESM model.

### Sensitivity of simulated surface temperature to ice cloud scattering properties

**Figure 3:**
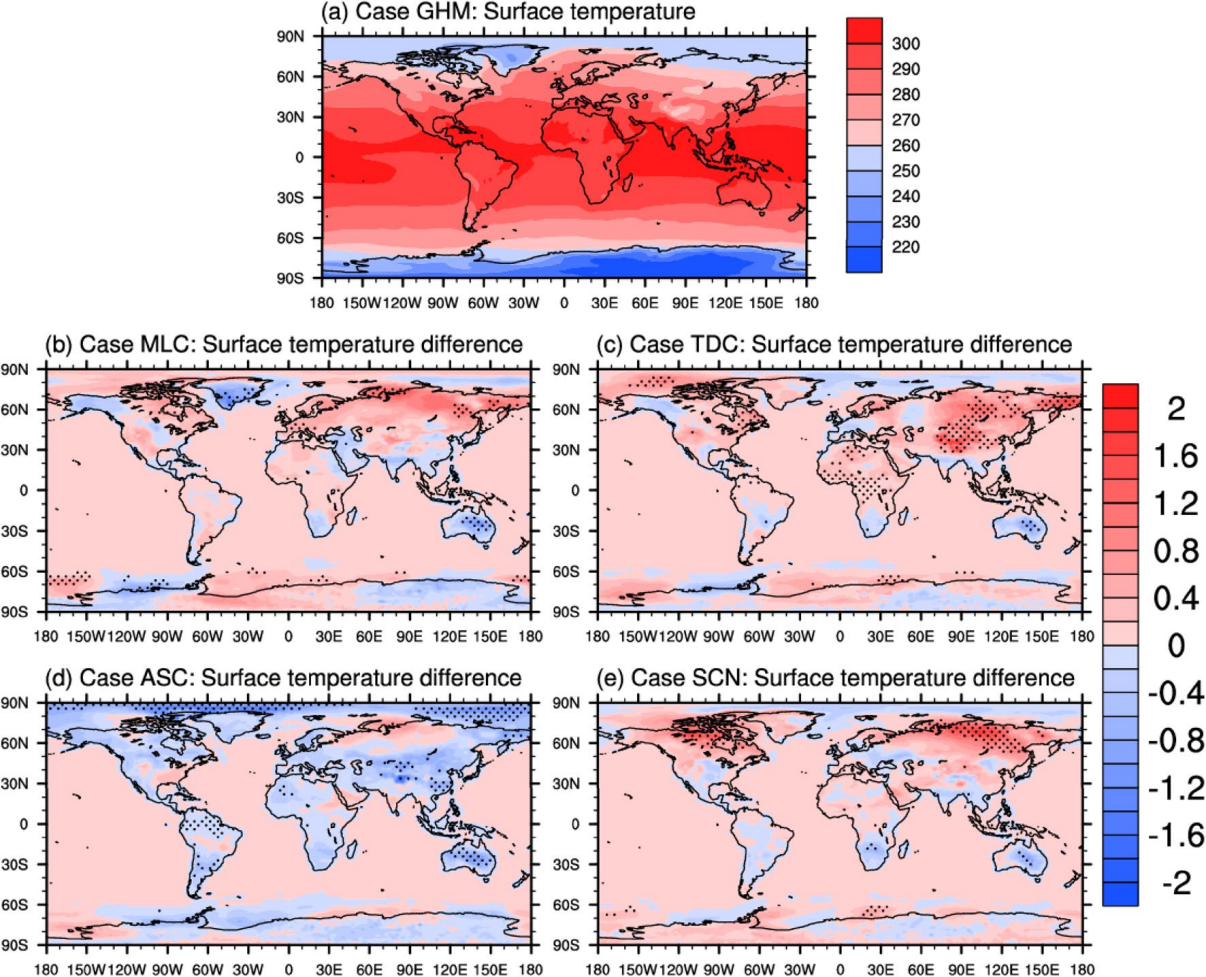
Simulated annual averaged surface temperature for the GHM case (**a**) and the corresponding differences between MLC, TDC, ASC, SCN and GHM cases (**b**–**e**). Unit: K. Stippled areas indicate significant differences at the 95% confidence level.

The combined effects of SWCRE and LWCRE are reflected in terms of the surface temperature changes in Fig. 3. The globally averaged net CREs can be found in Table 3 where very minor changes in net CREs are found between the MLC and SCN cases, while the TDC and ASC cases show positive and negative net CRE differences, respectively. As a result, different responses in the surface temperature are found for the five cases. Figure 3a shows the simulated surface temperature for the control case (GHM), and the panels (b)–(e) show the differences between the other cases and GHM. Significant changes of surface temperature are mostly found over land. And most of the differences in surface temperature correspond well with the differences in the net TOA CREs. For example, the surface temperature difference (STD) between the MLC and GHM is the lowest among all cases, where the negative STDs are found over Australia (contributed by the stronger SWCRE) and the positive STDs are found over high latitudes (contributed mostly by the LWCRE).

Although the globally averaged net CRE difference between SCN and GHM cases is almost zero, the STDs still show some features of positive patterns because the SWCRE and LWCRE differences unevenly distribute in the horizontal space. Generally, the STDs between SCN and GHM are similar as those between MLC and GHM except that broader areas with significant changes over North America and North Asia are found.

Broadly warmer surface temperature almost over all continental regions are found because significantly weaker SWCREs are globally found in the TDC case, except for Australia where particularly stronger SWCRE is present (Fig. 2e). The regional increase in the surface temperature can be more than 2 K over central and northeast Asia.

The ASC case shows much stronger SWCREs globally, especially over the tropical and midlatitude oceans. An overall decrease in the surface temperature is evident from Fig. 3d. Although stronger SWCREs occur over the southern oceans, such changes in the CRE are not sufficiently large to induce the perturbations in surface temperature locally. The surface temperature of the Arctic regions, however, show some sensitivities to the changes of ice cloud optical properties among the various simulations. It is anticipated that the differences in the surface condition and the reflectivity in the Arctic region could be contributing to the local STDs which requires further verification. It is worth noting that many climate models have relatively larger uncertainties in simulations over the Arctic region.

In summary, the most significant changes in surface temperature correspond well with the largest changes in the TOA CRE for all cases. However, it should be noted that there are several regions where the CRE differences and the STDs do not match. This indicates that the CRE is not the only driver of the STD, and the other factors should be considered to better understand the modeling results.

## Discussion

It is evident to find that the differences in the simulated surface temperature are mostly related to the differences in the local net CREs. However, the net CREs are determined not only by the cloud optical properties but also how the cloud particle sizes and cloud water are distributed horizontally and vertically. Considering that the simulated cloud fields among the five simulations are not significantly different (see Supplementary Figs. 4 and 5), our study could confirm and quantify the impacts of the ice cloud optical property parameterization on the simulation of cloud radiative effects and the surface temperatures.

Our study also answers the question of how different assumptions of ice cloud particle model could perturb the cloud radiative effects and the surface temperature simulations. The five ice cloud optical property parameterizations based on the five different ice cloud particle models are introduced under a same framework for quantitative estimations of different impacts from various factors. For example, the effects of ice habit mixture versus the single ice habit, the use of several ice habits featuring mid-latitude ice clouds versus those featuring tropical ice clouds, and the inclusion of ice surface roughness condition versus smooth single column ice habit, can be assessed through inter-comparing the five cases. Ice habit mixture and single-habit models (MLC versus SCN) do not necessarily give very different results in that their optical properties are similar and thus the simulated CREs and surface temperatures are also similar. Different ice habit mixtures focusing on different types of ice clouds (mid-latitude and tropical, MLC versus TDC) whereas result in quite different responses in the SWCREs where they are overestimated in the MLC case and are underestimated in the TDC case. A single-habit ice cloud model of ASC differs from the ice cloud model of SCN by resulting in much stronger SWCRE and relatively lower LWCRE.

In summary, we conclude that ice habit mixture models which are developed to be analogous to real ice clouds found at different regions of the globe could induce SWCRE difference within 1 Wm^−2^, but the difference in SWCRE could change the sign of number depending on the exact ice habits included in the ice model. A single-habit ice cloud particle model (ASC), which is found to be suitable for ice cloud property remote sensing retrievals, could induce additionally stronger SWCRE resulting in global surface cooling which amount to − 2 Wm^−2^ in SWCRE and several degrees of regional surface temperature reduction. Note our results of the CRE differences and the STDs are smaller than previous results like Zhao et al.^49^ This is because the previous studies mostly include more contributing factors like ice refractive indices and particle size distributions, which diverse remarkably among different ice optical property parameterizations. Given the model results depict quite different response in the simulations, special attentions should be exercised when different model simulations are compared, or when satellite observations and retrievals are used as truth for validation against model results. Additional hidden biases in cloud radiation effects are possibly influencing the climate feedbacks. Note also that if a coupled model is used, the impacts of ice cloud optical property parameterization over ocean could be intensified by the air-sea interaction and further induce greater changes in the surface temperature. Further studies about unifying the ice cloud scattering properties used in remote sensing and modeling communities are eagerly needed. There is still much work to be done to obtain full unification between climate models and remote sensing although some early attempts have been made^55^.

## Methods

### Ice cloud optical property parameterizations

The ice cloud optical property parameterization schemes are developed based on three multi-habit ice cloud particle models constructed by Baum et al.^26^ namely, General Habit Mixture (GHM), Midlatitude habit mixture (MLC), and Tropical Deep Convection (TDC), as well as two single-habit ice cloud particle models, namely, Aggregate of Severely roughened column (ASC) and Single Column (SCN) (see Table 1). A same set of ice cloud microphysical properties including particle size distributions (PSDs) derived from several field campaigns which are summarized to follow Gamma size distribution is used^56^. According to Baum et al.^26^, the GHM is based on all ice microphysical data regardless of the ice cloud temperature. The MLC is a mixture that is based on microphysical data that have cloud temperature above 213 K and below 233 K and that no aggregates of plates are included. The TDC is a mixture for the deep tropical convective ice clouds that do not include droxtals while a large fraction of aggregates of plates are included. The variation of ice habit fractions with maximum particle diameter of the three ice mixture particle models are shown in Supplementary Fig. 2.

There are ample discussions of what kinds of ice cloud particle models should be used for remote sensing retrieval and climate model simulations in the previous research studies. The selections of ice particle models here enable detailed examination of the impacts of various ice particle models with different complexities under a same framework. Specifically, the GHM can be regarded as the overall ice cloud model suitable for general and global use. The MLC and TDC, however, can be treated as the subsets of the GHM as they only focus on the midlatitude and the tropical deep convective ice clouds, respectively. The ASC is the same ice cloud model as that is used in the MODIS collection 6 ice cloud satellite retrieval. The SCN is an ice cloud model analogous to that of the Fu parameterization scheme^14^. Although previous study has found better spectral consistency for the ASC than the GHM^2^, the GHM still can be regarded as the controlled case with sufficient complexity in this study. Thus, comparisons of the MLC, TDC, ASC, and SCN cases to the controlled case (GHM) could reveal different implications of the influences of ice cloud complexities on simulated climate.

We follow the same approach as is described in CESM/CAM5 technical documentation^57^ to develop the five ice optical property parameterization schemes and to implement them in the model experiments. Single scattering properties of ice crystals are derived from a comprehensive database developed by Yang et al.^58^ and Bi and Yang^59^ using the latest light-scattering computation techniques of Invariant Imbedding T-matrix Method and Improved Geometric Optics Method^60^ in which a same set of ice refractive indices are used in the calculations. The bulk ice scattering properties used in CAM5 including the mass extinction and absorption coefficients, single-scattering albedo, and asymmetry factor, are parameterized as functions of the ice cloud particle effective diameter in the range of 10–120 μm. The effective diameter^26,61^ is defined as:

1 $$D_{eff} = \frac{{3\mathop \sum \nolimits_{i = 1}^{i = M} \left[ {\mathop \smallint \nolimits_{{D_{\min } }}^{{D_{\max } }} V_{i} \left( D \right)f_{i} \left( D \right)N\left( D \right)dD} \right]}}{{2\mathop \sum \nolimits_{i = 1}^{i = M} \left[ {\mathop \smallint \nolimits_{{D_{\min } }}^{{D_{\max } }} A_{i} \left( D \right)f_{i} \left( D \right)N\left( D \right)dD} \right]}},$$

where ${D}_{eff}$ is the ice cloud effective diameter, *D* is the single ice particle maximum dimension, *D*_*min*_ and *D*_*max*_ are the lower and upper limits of the ice particle dimension, *A*_*i*_ and *V*_*i*_ are the projected area and volume of the single ice particle with the ice shape *i*, *f*_*i*_ is the fraction of ice shape *i* which varies with the size of ice particle (see Supplementary Fig. 2), *M* is the total number of ice shapes in a particular ice cloud particle model, and *N* is the PSD.

The equations to derive the bulk scattering properties of ice cloud are shown below following previous study^47^:

2 $$k_{ext} = \frac{{\mathop \smallint \nolimits_{{\lambda_{\min } }}^{{\lambda_{\max } }} \mathop \smallint \nolimits_{{D_{\min } }}^{{D_{\max } }} \mathop \sum \nolimits_{i = 1}^{i = M} Q_{ext,i} \left( {D,\lambda } \right)A_{i} \left( D \right)f_{i} \left( D \right)N\left( D \right)S\left( \lambda \right)dDd\lambda }}{{\mathop \smallint \nolimits_{{\lambda_{\min } }}^{{\lambda_{\max } }} \mathop \smallint \nolimits_{{D_{\min } }}^{{D_{\max } }} \mathop \sum \nolimits_{i = 1}^{i = M} V_{i} \left( D \right)f_{i} \left( D \right)N\left( D \right)S\left( \lambda \right)dDd\lambda }},$$

3 $$\omega = \frac{{\mathop \smallint \nolimits_{{\lambda_{\min } }}^{{\lambda_{\max } }} \mathop \smallint \nolimits_{{D_{\min } }}^{{D_{\max } }} \mathop \sum \nolimits_{i = 1}^{i = M} \sigma_{sca,i} \left( {D,\lambda } \right)f_{i} \left( D \right)N\left( D \right)S\left( \lambda \right)dDd\lambda }}{{\mathop \smallint \nolimits_{{\lambda_{\min } }}^{{\lambda_{\max } }} \mathop \smallint \nolimits_{{D_{\min } }}^{{D_{\max } }} \mathop \sum \nolimits_{i = 1}^{i = M} \sigma_{ext,i} \left( {D,\lambda } \right)f_{i} \left( D \right)N\left( D \right)S\left( \lambda \right)dDd\lambda }},$$

4 $$g = \frac{{\mathop \smallint \nolimits_{{\lambda_{\min } }}^{{\lambda_{\max } }} \mathop \smallint \nolimits_{{D_{\min } }}^{{D_{\max } }} \mathop \sum \nolimits_{i = 1}^{i = M} g_{i} \left( {D,\lambda } \right)\sigma_{sca,i} \left( {D,\lambda } \right)f_{i} \left( D \right)N\left( D \right)S\left( \lambda \right)dDd\lambda }}{{\mathop \smallint \nolimits_{{\lambda_{\min } }}^{{\lambda_{\max } }} \mathop \smallint \nolimits_{{D_{\min } }}^{{D_{\max } }} \mathop \sum \nolimits_{i = 1}^{i = M} \sigma_{sca,i} \left( {D,\lambda } \right)f_{i} \left( D \right)N\left( D \right)S\left( \lambda \right)dDd\lambda }},$$

5 $$k_{abs} = \frac{{\mathop \smallint \nolimits_{{\lambda_{\min } }}^{{\lambda_{\max } }} \mathop \smallint \nolimits_{{D_{\min } }}^{{D_{\max } }} \mathop \sum \nolimits_{i = 1}^{i = M} Q_{abs,i} \left( {D,\lambda } \right)A_{i} \left( D \right)f_{i} \left( D \right)N\left( D \right)S\left( \lambda \right)dDd\lambda }}{{\mathop \smallint \nolimits_{{\lambda_{\min } }}^{{\lambda_{\max } }} \mathop \smallint \nolimits_{{D_{\min } }}^{{D_{\max } }} \mathop \sum \nolimits_{i = 1}^{i = M} V_{i} \left( D \right)f_{i} \left( D \right)N\left( D \right)S\left( { \lambda } \right)dDd\lambda }},$$

where ${k}_{ext}$, $\omega$, $g$, and ${k}_{abs}$ are the bulk mass extinction coefficient, single-scattering albedo, asymmetry factor, and mass absorption coefficient, ${\lambda }_{max}$ and ${\lambda }_{min}$ are the upper and lower wavelength limits of the radiation bands, ${\sigma }_{sca,i}$ and ${\sigma }_{ext,i}$ are the scattering and extinction cross section for the ice shape *i*, ${Q}_{ext,i}$ and ${Q}_{abs,i}$ are the extinction and absorption efficiencies for the ice shape *i*, ${g}_{i}$ is the asymmetry factor for the ice shape *i*, and *S* is the solar spectrum for SW bands and is otherwise replaced by the Planck function at 233 K for the LW bands.

### The CAM5 model simulations and experiments

The CAM5.3 model is the atmospheric component of the CESM (Community Earth System Model) version 1.2.2 developed by NCAR (National Center for Atmospheric Research, USA). In this study, we carry out five numerical experiments with CAM5.3 implementing five different ice cloud optical property parameterization schemes respectively to replace the default scheme^29^. Each experiment is interactively run for 70 years to climate equilibrium with the same initial state of the year 2000 driven by cyclic annual variations of sea surface temperature at 1.9° × 2.5° finite volume grid resolution. The CAM5 physics are used and the COSP (CFMIP Observational Simulator Package) cloud diagnostics^62^ are enabled in the simulations to provide suitable simulation results for comparison with satellite retrievals of cloud properties. Only the last 10 years of simulation results are analyzed with the Atmosphere Model Working Group diagnostics package for diagnostic purposes. The GHM simulation case is treated as the controlled experiment and the other cases are compared with the controlled experiment to discern the various features associated with the optical property changes. Also note that the longwave cloud scattering effect is not considered in the radiative transfer modeling in CESM which may induce some biases in the longwave cloud radiative effect^63–65^.

## Supplementary Information

Supplementary Information.

## Data Availability

All codes and data used to derive the results reported in this study are available upon request. The CESM model is available at http://www.cesm.ucar.edu/models.
