# Supplementary material for: Diverse cloud radiative effects and global surface temperature simulations induced by different ice cloud optical property parameterizations
Source: Sci Rep. 2022 Jun 22;12:10539. doi: 10.1038/s41598-022-14608-w (PMC9217801; doi:10.1038/s41598-022-14608-w)
Supplement: Supplementary file 1 — Supplementary Information. [file 41598_2022_14608_MOESM1_ESM.docx]

Supplementary Materials for

**Diverse cloud radiative effects and global surface temperature simulations induced by different ice cloud optical property parameterizations**

Bingqi Yi*

*Corresponding author. Email: yibq@mail.sysu.edu.cn

This PDF file includes:

Supplementary Figure 1. Ice cloud optical property differences as functions of the ice particle effective diameter for various SW and LW bands.

Supplementary Figure 2. Ice habit fractions of the GHM (upper left panel), MLC (upper right panel), and TDC (lower left panel) cases following Baum et al. (2011) with minor updates in the ice habit fractions.

Supplementary Figure 3. Model simulated averaged ice cloud properties for the GHM case.

Supplementary Figure 4. Zonally averaged cloud liquid water (upper left panel) and ice (lower left panel) water mixing ratios (Unit: g/kg) for the GHM case, and the differences in liquid water (upper right panel) and ice (lower right panel) mixing ratios between the ASC and GHM cases. The other cases are similar and thus are not shown.

Supplementary Figure 5. Model simulated averaged ice cloud particle effective radius for the GHM case (a) and the corresponding differences between the MLC, TDC, ASC, SCN and the GHM (b-e). Unit: μm.

Supplementary Figure 6. Ten-year (March 2000 to February 2010) annual averaged CERES EABF SW and LW cloud radiative effects (a, b) and the corresponding differences between the GHM, MLC, TDC, ASC, and SCN cases and the CERES EBAF (Edition 4.1) (c-l). The left column panels are the shortwave CREs and the right column panels are the longwave CREs. Unit: Wm^-2^.


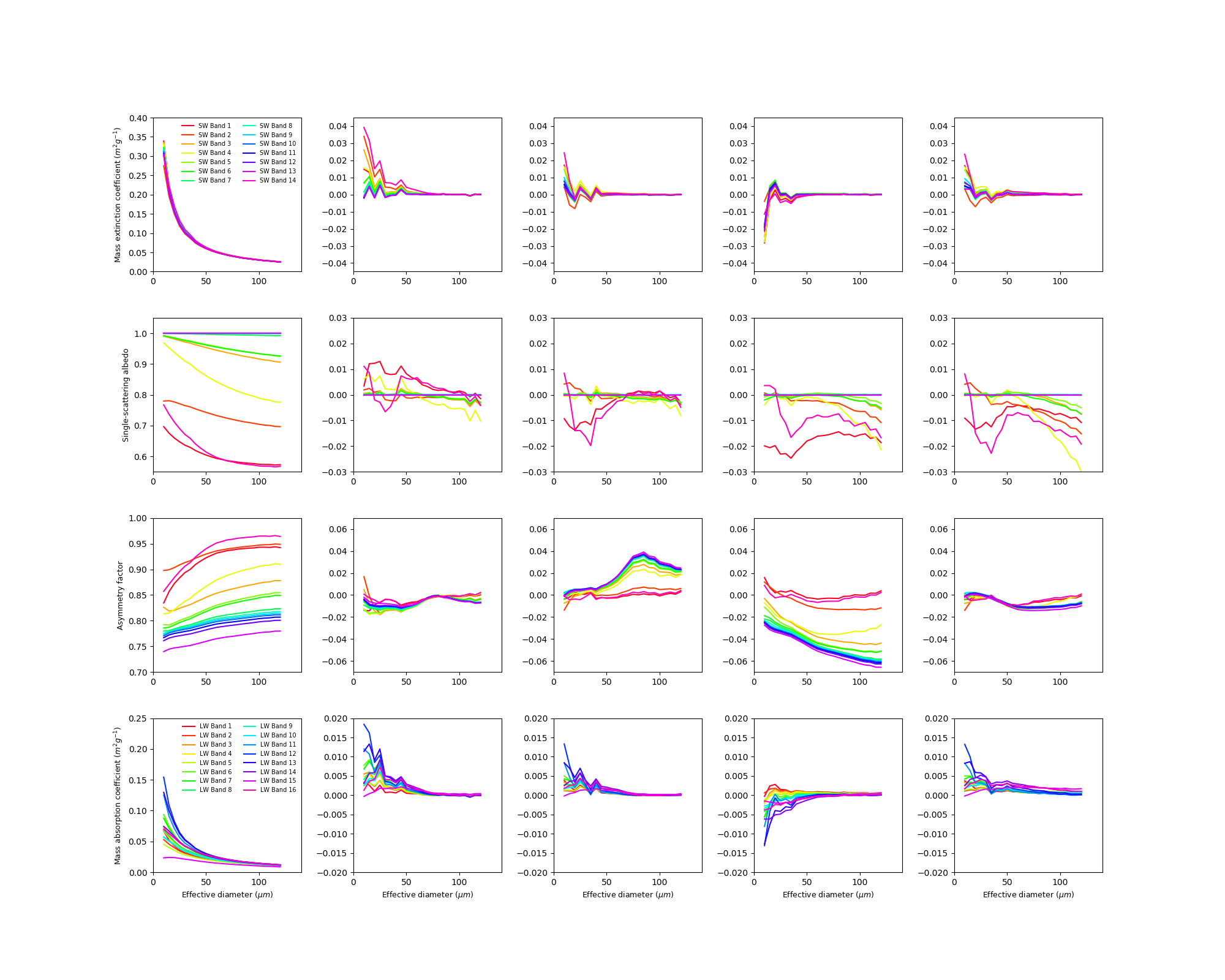


Supplementary Fig. 1 Ice cloud optical property differences as functions of the ice particle effective diameter for various SW and LW bands. Rows from top to bottom are the mass extinction coefficient, single-scattering albedo, asymmetry factor, and mass absorption coefficient. Columns from left to right are the GHM, MLC minus GHM, TDC minus GHM, ASC minus GHM, and SCN minus GHM cases. The corresponding SW and LW bands are the same as Figure 1.


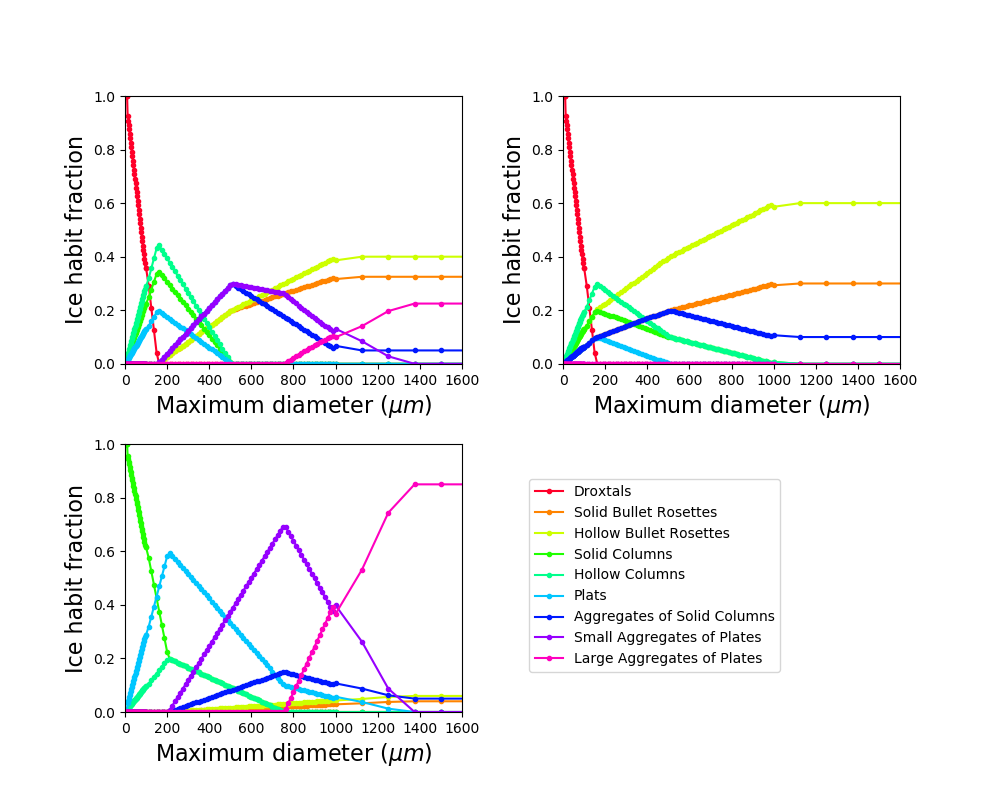


Supplementary Fig. 2 Ice habit fractions of the GHM (upper left panel), MLC (upper right panel), and TDC (lower left panel) cases following Baum et al. (2011) with minor updates in the ice habit fractions.


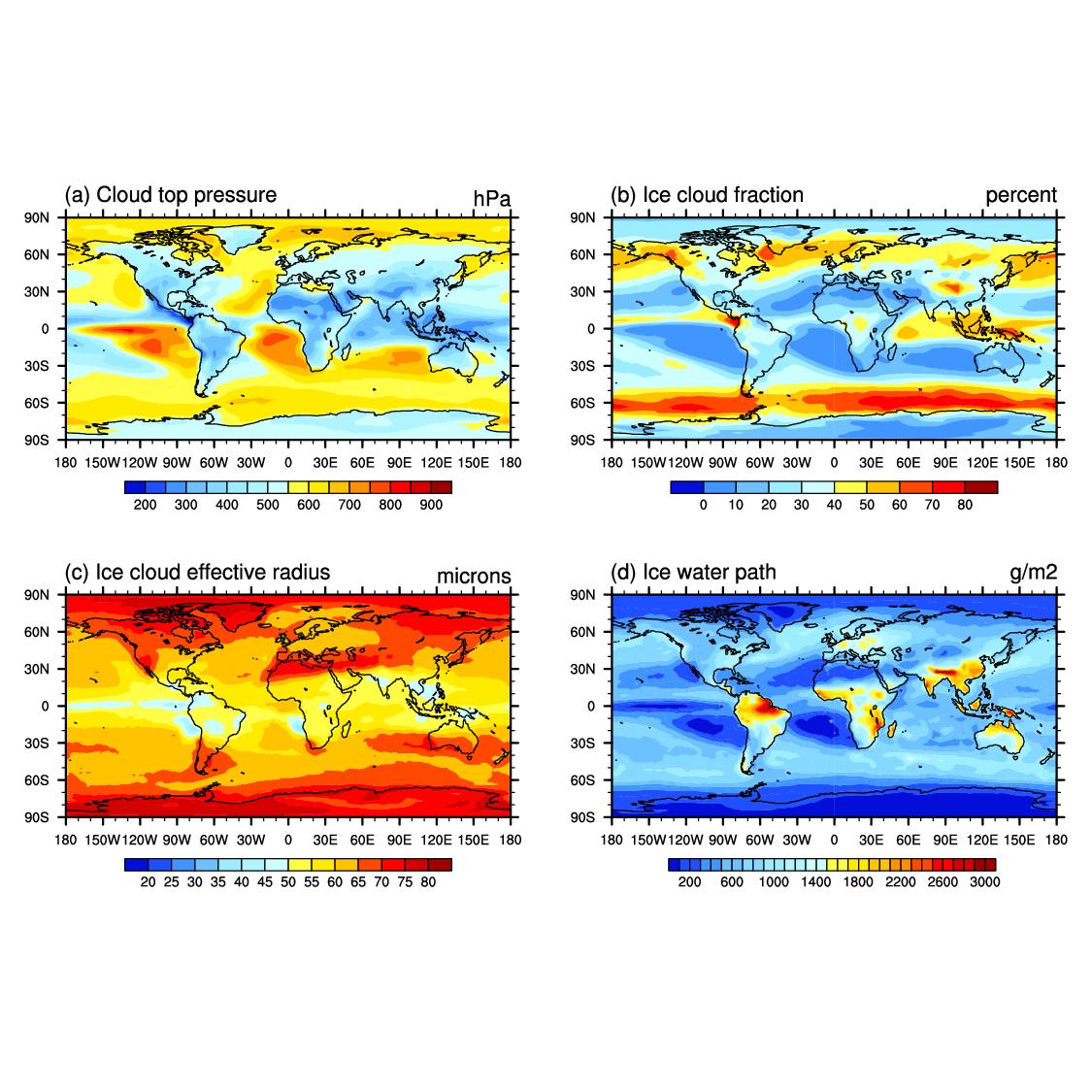


Supplementary Fig. 3 Model simulated averaged ice cloud properties with COSP cloud diagnostics for the GHM case. (a) cloud top pressure; (b) ice cloud fraction; (c) Ice cloud effective radius; (d) Ice water path.


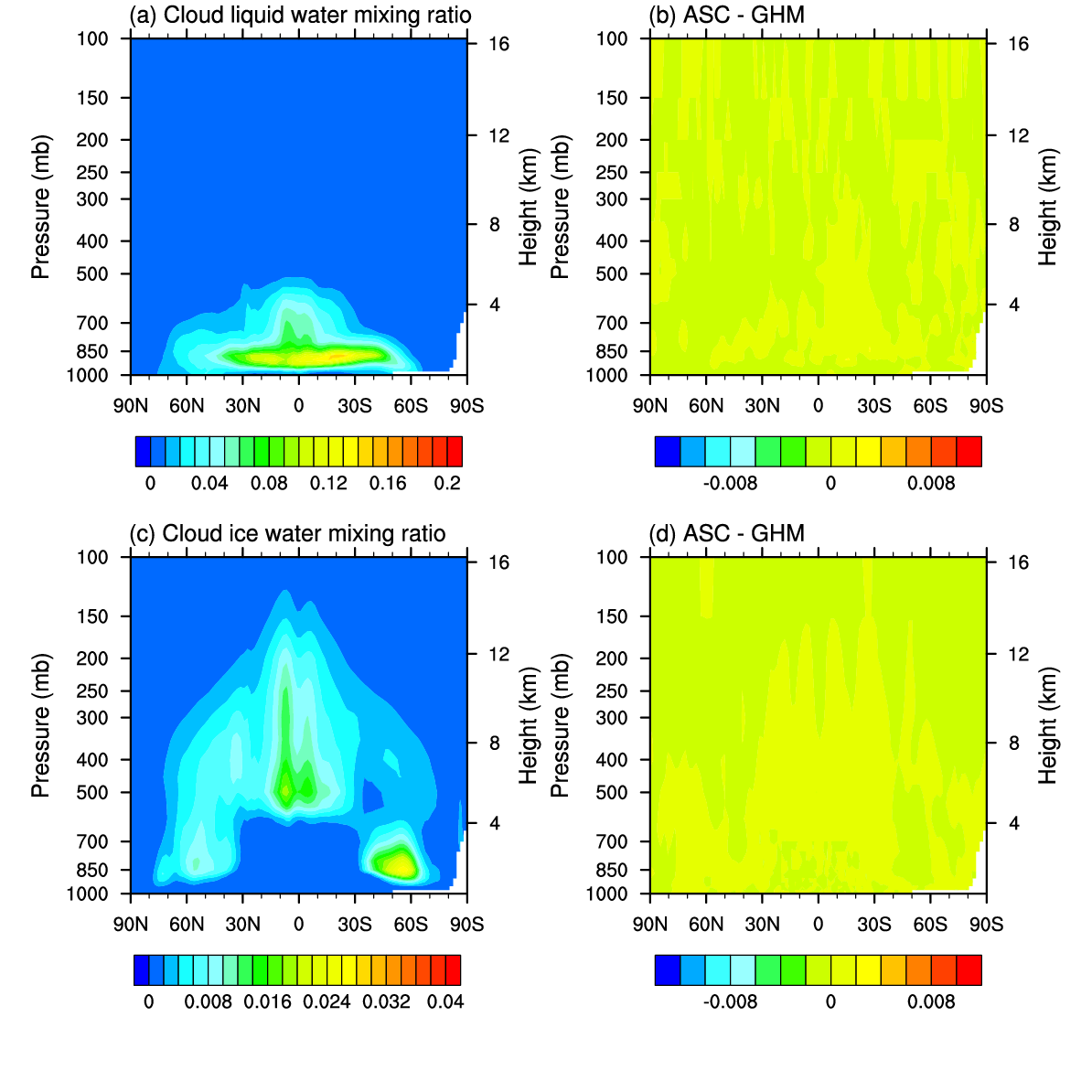


Supplementary Fig. 4 Zonally averaged cloud liquid water (upper left panel) and ice (lower left panel) water mixing ratios (Unit: g/kg) for the GHM case, and the differences in liquid water (upper right panel) and ice (lower right panel) mixing ratios between the ASC and GHM cases. The other cases are similar and thus are not shown.


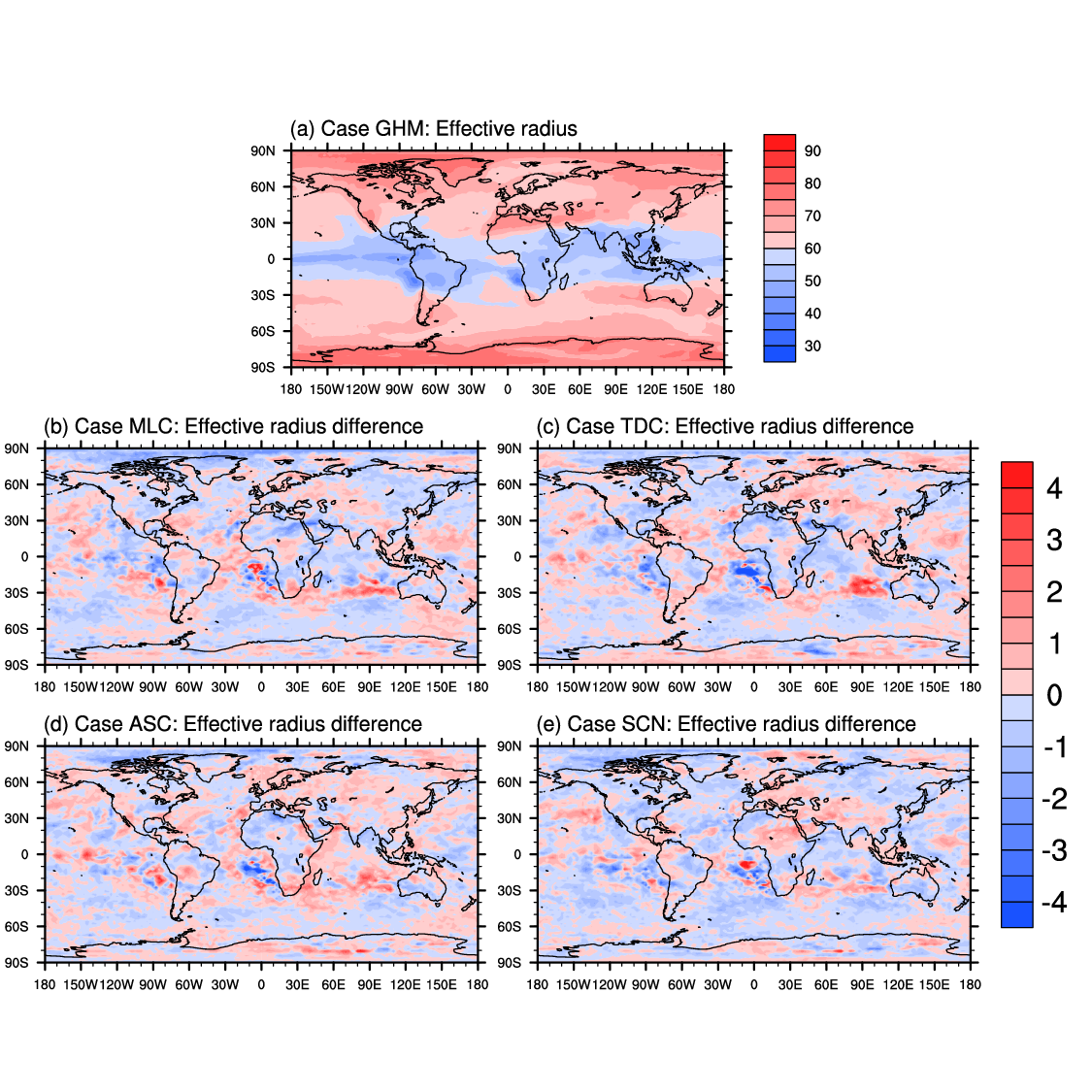


Supplementary Fig. 5 Model simulated averaged ice cloud particle effective radius for the GHM case (a) and the corresponding differences between the MLC, TDC, ASC, SCN and the GHM (b-e). Unit: μm.


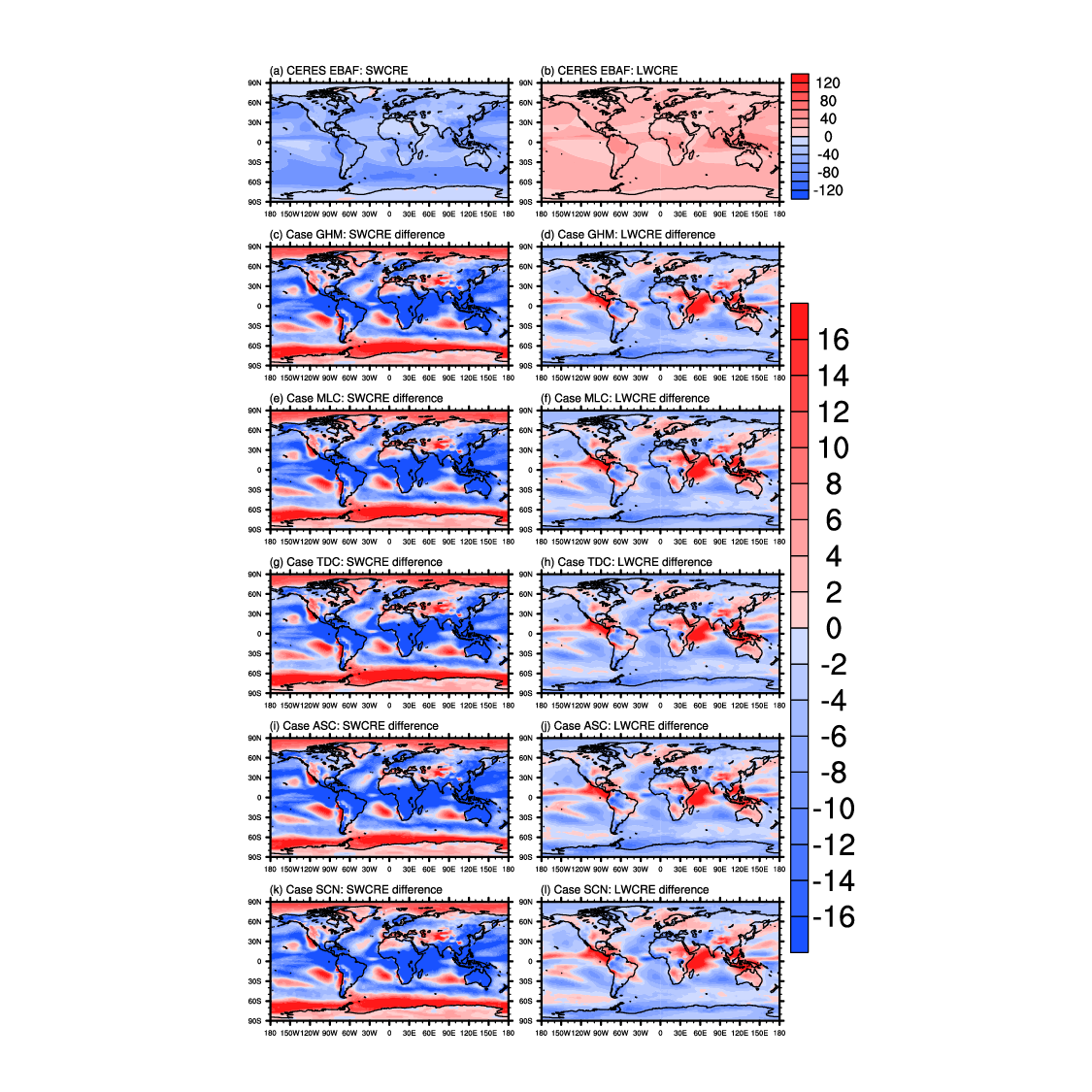


Supplementary Fig. 6 Ten-year (March 2000 to February 2010) annual averaged CERES EABF SW and LW cloud radiative effects (a, b) and the corresponding differences between the GHM, MLC, TDC, ASC, and SCN cases and the CERES EBAF (Edition 4.1) (c-l). The left column panels are the shortwave CREs and the right column panels are the longwave CREs. Unit: Wm^-2^.
